# RETRACTION: Effects of Allogenic Acellular Dermal Matrix Combined With Autologous Razor‐Thin Graft on Hand Appearance and Function of Patients With Extensive Burn Combined With Deep Hand Burn

**DOI:** 10.1111/iwj.70651

**Published:** 2025-04-23

## Abstract

**RETRACTION:** ShangF. and HouQ. , “Effects of Allogenic Acellular Dermal Matrix Combined With Autologous Razor‐Thin Graft on Hand Appearance and Function of Patients With Extensive Burn Combined With Deep Hand Burn,” International Wound Journal 18, no. 3 (2021): 279–286, 10.1111/iwj.13532.33547855 PMC8243990

The above article, published online on 6 February 2021, in Wiley Online Library (http://onlinelibrary.wiley.com/), has been retracted by agreement between the journal Editor in Chief, Professor Keith Harding; and John Wiley & Sons Ltd. Following an investigation by the publisher, all parties have concluded that this article was accepted solely on the basis of a compromised peer review process. In addition, further investigation by the publisher found that the authors provided incomplete ethical approval information for the study. The editors have therefore decided to retract the article. The authors did not respond to our notice regarding the retraction.

**RETRACTION:** F. Shang and Q. Hou , “Effects of Allogenic Acellular Dermal Matrix Combined With Autologous Razor‐Thin Graft on Hand Appearance and Function of Patients With Extensive Burn Combined With Deep Hand Burn,” International Wound Journal 18, no. 3 (2021): 279–286, 10.1111/iwj.13532.33547855 PMC8243990

The above article, published online on 6 February 2021, in Wiley Online Library (http://onlinelibrary.wiley.com/), has been retracted by agreement between the journal Editor in Chief, Professor Keith Harding; and John Wiley & Sons Ltd. Following an investigation by the publisher, all parties have concluded that this article was accepted solely on the basis of a compromised peer review process. In addition, further investigation by the publisher found that the authors provided incomplete ethical approval information for the study. The editors have therefore decided to retract the article. The authors did not respond to our notice regarding the retraction.

